# Adsorption-based membranes for air separation using transition metal oxides†

**DOI:** 10.1039/d1na00307k

**Published:** 2021-06-25

**Authors:** Asmita Jana, David S. Bergsman, Jeffrey C. Grossman

**Affiliations:** Department of Materials Science and Engineering, Massachusetts Institute of Technology 77 Massachusetts Avenue Cambridge Massachusetts 02139 USA jcg@mit.edu

## Abstract

In this work, we use computational modeling to examine the viability of adsorption-based pore-flow membranes for separating gases when a purely size-based separation strategy is ineffective. Using molecular dynamics simulations of O_2_ and N_2_, we model permeation through a nanoporous graphene membrane. Permeation is assumed to follow a five-step adsorption-based pathway, with desorption being the rate-limiting step. Using this model, we observe increased selectivity between O_2_ and N_2_, resulting from increased adsorption energy differences. We explore the limits of this strategy, providing an initial set of constraints that need to be satisfied to allow for selectivity. Finally, we provide a preliminary exploration of some transition metal oxides that appear to satisfy those conditions. Using density functional theory calculations, we confirm that these oxides possess adsorption energies needed to operate as adsorption-based pore-flow membranes. These adsorption energies provide a suitable motivation to examine adsorption-based pore-flow membranes as a viable option for air separation.

## Abstract 2

Adsorption energy differences can lead to selectivity.

## Graphical abstract

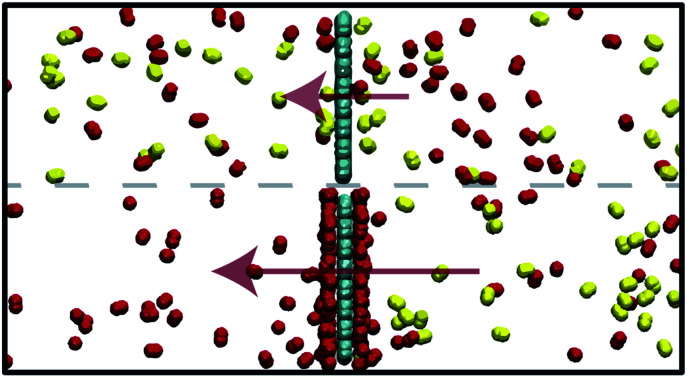

## Introduction

Massive volumes of industrial grade oxygen (O_2_ concentration > 99.5%) are employed in wide-ranging industries, from automotive to healthcare to metal manufacturing.^1^ In particular, it is important in industries such as steel, aluminum, copper, glass, pulp and paper, petroleum, and power production that use oxy-fuel combustion.^2^ Pure oxygen not only eliminates formation of toxic nitrogen oxides from nitrogen contamination but also leads to easier carbon dioxide capture and recovery, due to higher concentrations of CO_2_ in the exhaust.^3^ For example, the production of methanol, lime, and sodium carbonate used around 320 TBtu in 2001 and employing oxygen enriched air (OEA) instead of air can lead to energy savings of around 5 TBtu per year which corresponds to around 1.5% of the energy used in those processes.^4^ While OEA is already used in a range of industries, it remains relatively expensive to produce, in terms of energy, which can potentially outweigh the energy savings associated with it.

Currently, the most common process used to produce pure oxygen and nitrogen, cryogenic distillation, requires 774 Btu per 1 lb of nitrogen and oxygen.^5^ Because roughly 70 billion lb of nitrogen gas is produced annually, this requires 54 TBtu per year which corresponds to around 0.6% of the total US energy consumption.^4^ This energy is consumed primarily in the form of cryogenic distillation, of which 91% is consumed by compressors. Although any improvement in this process could lead to large energy savings, rather than try to slightly improve the already well-optimized distillation process, switching to alternative separation strategies has the potential of reducing the overall energy requirement by 50%, which would save around 23 TBtu per year.^4^ Many alternative separation strategies exist, such as Pressure Swing Adsorption (PSA), where adsorbents selectively bind certain component(s) of the mixture at high pressure.^6^ When the pressure is lowered, the adsorbed species desorb. However, the adsorption and desorption process inevitably reduce energy efficiency and prevents the use of a continuous process.

Membrane separations are a promising alternative to cryogenic distillation and batch-type separations like PSA. It has been suggested that a major fraction of the energy (10 TBtu per year), which would otherwise be consumed in liquefaction, could be saved if membrane separations were used for air separation instead.^4^ However, the use of membranes for air separation is limited primarily in the materials design of the membranes themselves. For instance, polymer membranes, which have conventionally been used for air separations, produce high purity nitrogen. While O_2_ permeates faster through the membrane than N_2_ due to its high diffusivity and smaller size, a small quantity of N_2_ also inevitably diffuses through. This results in OEA in the permeate while the recovered feed gas is highly concentrated N_2_ gas. Thus, these membranes are used to obtain high purity N_2_ gas and a pure stream of O_2_ is relatively harder to obtain using a polymer membrane.^7,9^ Some of the polymers used for O_2_/N_2_ separation include polysulfone (PSU), polyimide (Matrimid), and poly(2,6-dimethyl-1,4-phenylene oxide) (PPO).^10^ Polymers of intrinsic microporosity (PIMs) are a class of materials extensively used for O_2_/N_2_ separation due to its high O_2_ permeability.^11^ PIM-1 displays O_2_ permeability of 700–1700 Barrer with O_2_/N_2_ selectivity of around 2.2–4.3.^12–17^ However, most polymer membranes are limited by the Robeson upper limit.^8^ The Robeson upper limit describes an empirical upper bound inverse relationship between selectivity and permeability observed for gas permeation across polymer membranes.^8^ To overcome the upper limit, they are combined with metal–organic frameworks (MOFs) and the resultant mixed matrix membranes (MMMs) display enhanced performance.^18–20^ One such MMM, the PIM-1/ZIF-8-7 composite fabricated by Liu *et al.*^21^ displays O_2_ permeability of 1287 Barrer and O_2_/N_2_ selectivity of 3.7. Moreover, selectivity of PIM-1 membranes was enhanced by converting it to a molecular sieve membrane through an intermediate thermal treatment.^22^ Polyimides like Matrimid are another class of materials also used for O_2_/N_2_ separations with O_2_/N_2_ selectivity of around 6–6.6.^23–25^ Various composites of Matrimid, like Matrimid/polyethersulfone (PES), and Matrimid/P84 (BTDA-TDI/MDI, co-polyimide of 3,3′,4,4′-benzophenone tetracarboxylic dianhydride and 80% methylphenylene-diamine + 20% methylene diamine) result in enhanced selectivity.^26,27^ When combined with PIM-1, a 3.5-fold higher O_2_ permeance was observed compared to pristine Matrimid.^28^ Facilitated transport is another strategy used where O_2_ permeates *via* a hopping mechanism, where O_2_ molecules jump from one carrier to another by selective and reversible complexation.^29^ Co-based complexes, like cobalt(ii) phthalocyanine (CoPC), cobalt(ii) tetraphenylporphyrin (CoTPP), and cobalt(iii) acetylacetonate (Co(acac)_3_) demonstrate high O_2_ selectivity.^29–33^ Incorporation of Pluronic-treated cobalt(ii) phthalocyanine microparticles (CoPCMPs) to Matrimid results in O_2_/N_2_ selectivity of around 7.8 and O_2_ permeability of 2.82 Barrer.^34^

Since polymer membranes operate under the solution-diffusion mechanism, they are limited by the permeability-selectivity trade-off as depicted by the Robeson upper bound.^8^ However, pore-flow membranes are not limited by this bound. N_2_/O_2_ separation using pore-flow membrane technology is challenging due to their similar sizes and masses. These membranes typically rely on molecular sieving and Knudsen diffusion as the major mechanisms of separation, governed by size and mass, respectively. For oxygen and nitrogen, the kinetic diameters are 0.346 nm and 0.364 nm, respectively, rendering any membrane that relies solely on separation through size and mass highly limited in selectivity. Thus, a distinct need to obtain higher selectivity motivates “out-of-the-box” ideas for improved O_2_/N_2_ separation membranes.

Computational tools are increasingly playing an important role in the study of membrane design and performance because of the myriad advantages, including the ability to probe atomic-level phenomena, analyse parameter-space that is otherwise difficult to access experimentally, gather insights into and isolate the fundamental physics responsible for a given property, and subsequently tune it by designing optimized membranes aimed at maximizing performance.

In this paper, we employed computational tools to investigate the use of membranes with explicit pores that rely on the pore-flow mechanism rather than the solution-diffusion mechanism, to push permeability and selectivity beyond the Robeson upper limit.^8^ In particular, we are interested in exploring the selectivity generated by a difference in adsorption energies, and the limits of the resulting selectivity and what governs them. We employed molecular dynamics (MD) simulations to artificially tune the adsorption energy of oxygen gas onto a nanoporous graphene membrane while keeping that of nitrogen fixed. The hypothetical gases were then allowed to permeate through the membrane with pores large enough to ensure both gases can pass and the selectivity was calculated. We observed an increase in selectivity arising solely from increased adsorption energy differences, demonstrating the use of adsorption as a viable mechanism for separating O_2_ from N_2_. From these MD simulations it was possible to develop an initial set of constraints on these adsorption energy differences that would need to be satisfied to allow for selectivity. Using density functional theory (DFT), we calculated the adsorption energies for Fe_2_O_3_ and Co_3_O_4_ in order to show example materials that satisfy the constraints placed by this strategy. The theoretical permeability and selectivity for the Fe_2_O_3_ and Co_3_O_4_ pore-flow membranes are shown to lay beyond the Robeson upper limit,^8^ motivating further study of membranes based on this principle.

## Direct and adsorbed-phase pathway

There are two reported pathways through which gas permeates through a porous membrane—the direct-phase pathway and the adsorbed-phase pathway.^35–37^ Usually, both pathways are active, and their contribution to the total flux is a function of temperature, pressure, adsorption energies, and size differences between the permeating species.^36^ The direct-phase pathway corresponds to molecules permeating through pores as a gas, without first adsorbing to the porous surface. For this pathway, the driving force for permeation is the difference in partial pressures and the resulting flux is given by eqn (1).^36^1
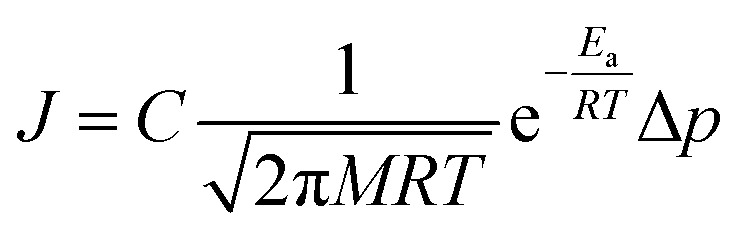
Here, *J* represents flux, *M* is the molecular mass, *T* is the temperature, *R* is the gas constant, *E*_a_ is the activation energy, Δ*p* is the difference in partial pressures and *C* is a geometric constant accounting for the shape of the pores.

**Fig. 1:**
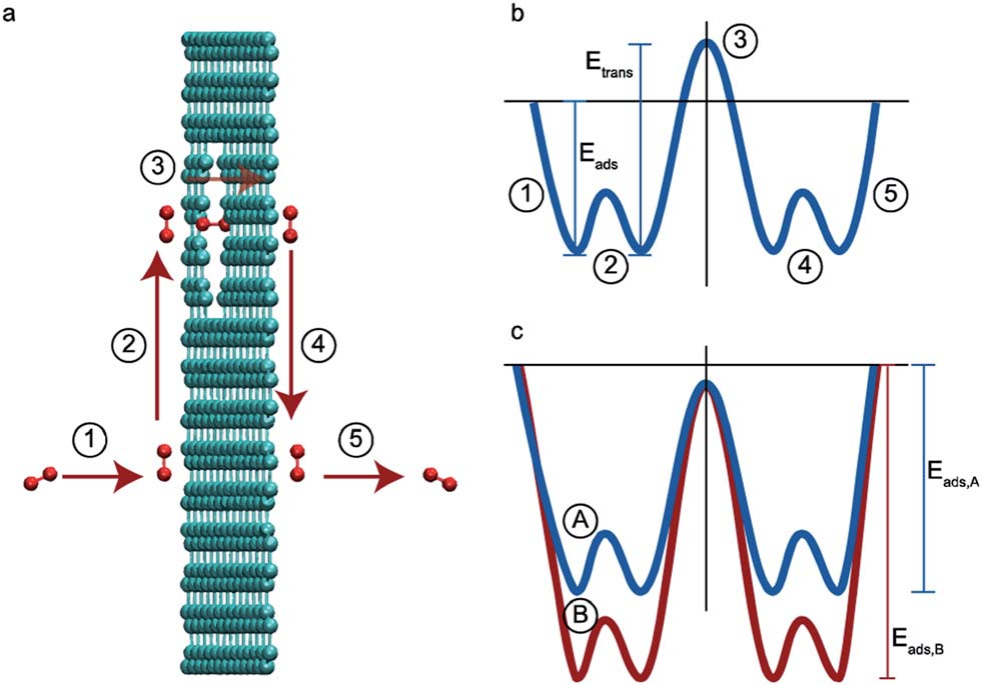
(a) The five elementary steps in an adsorbed-phase pathway, (1) adsorption, (2) association, (3) translocation, (4) dissociation, and (5) desorption. (b) The energy barriers for the five elementary steps. Adapted from ref. 36. (b) Energy barriers for two species A and B where B is adsorbed preferentially over A and the desorption step is the rate determining one for the species.

The adsorbed-phase pathway involves species adsorbing to the membrane surface before translocating through the membrane. Unlike the direct-phase pathway, this process consists of five steps – adsorption, association, translocation, dissociation and desorption.^36,37^ Each of these have their own fluxes and activation energies. A typical energy profile is shown in Fig. 1b. The slowest step has the highest activation energy and determines the overall rate.

In size-based sieving, the translocation step is often the rate determining factor that ultimately leads to selectivity.^36^ Due to steric effects, bigger species tend to have a much higher activation energy compared to smaller species and thus experience drastically hindered permeation across the membrane. In the case of gases with similar sizes and masses, these translocation rates are the same. Thus, size-based separations have historically not been a viable option for gases. To overcome this limitation in new membrane designs, the rate limiting step must be shifted from the translocation step to another step along this permeation pathway.

### Desorption as a rate limiting step

In this paper, we explore computationally the use of an adsorption-based membrane with desorption as the rate determining step.

**Fig. 2:**
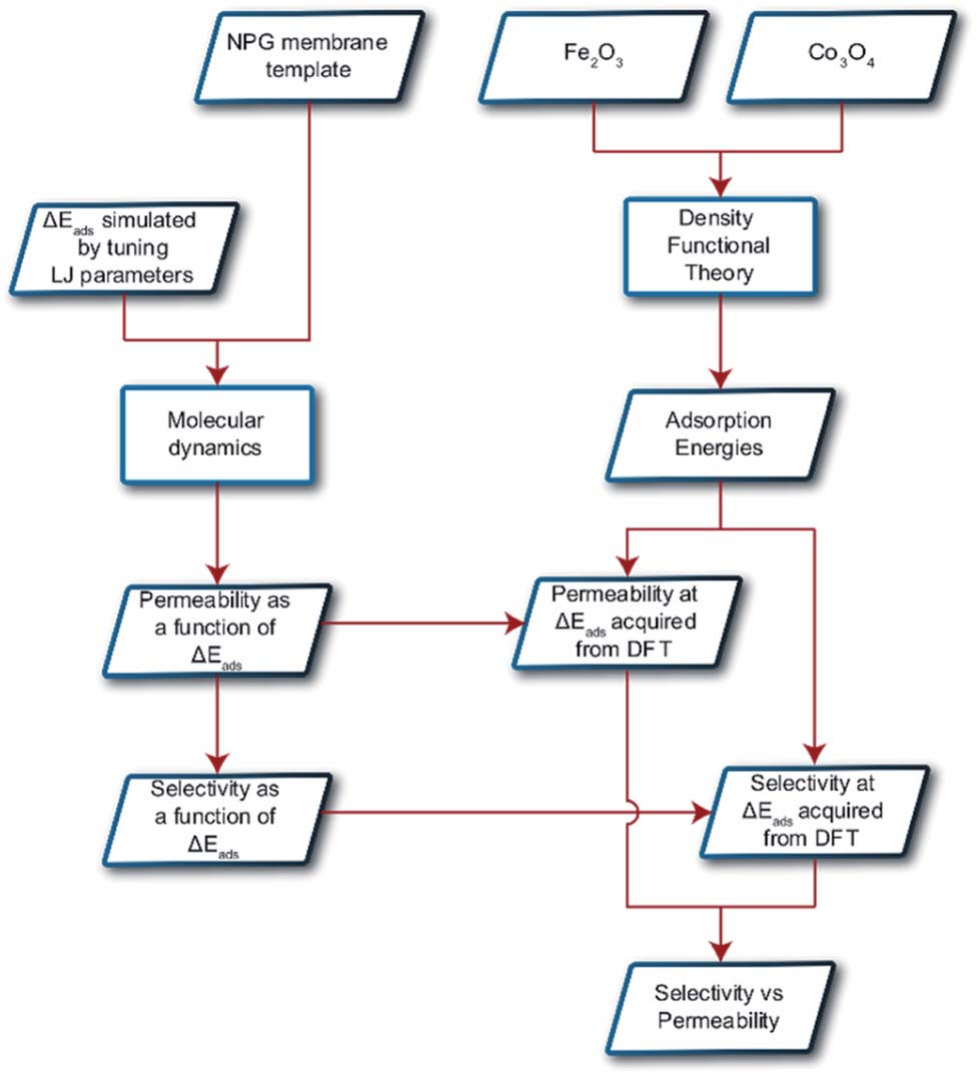
Schematic displaying the overall procedure incorporated and the simulation techniques employed in this work. Parallelograms represent input/output and rectangles represent methodologies.

Consider two gases A and B permeating through a membrane such that their energy profile looks like the one shown in Fig. 1c. The rate determining step for both gases is desorption, and the difference in the adsorption energies would lead to a difference in flux and selectivity. This idea has been proposed before as selective surface flow (SSF™) membranes for gas mixtures like hydrogen and hydrocarbons, and the resulting adsorption competition can drive selectivity.^38–40^ The selectivity was shown to arise from the preferential adsorption of one component over the other and the subsequent surface diffusion of the more strongly adsorbed component. Moreover, the preferentially adsorbed molecules on the pore walls also hinders the diffusion (in addition to the adsorption) of the other species, resulting in increased selectivity. Many membranes have been experimentally designed for various gas separations, but to design better membranes, a comprehensive understanding of how selectivity changes with adsorption energy differences is crucial. While the selectivity is highly dependent on the membrane material as well as the gas species chosen for separation, there are underlying trends and constraints that can be generalized. Thus, further work to study the dependence of selectivity on adsorption energy differences could lead us to understand the limits of these membranes and facilitate in the synthesis of membranes with improved performance. Below, we calculate the selectivity that would be obtained from these membranes and the limit of selectivity as a function of the difference in adsorption energies of the two gases onto the membrane surface (Fig. 2).

## Computational methods

### Molecular dynamics

**Fig. 3:**
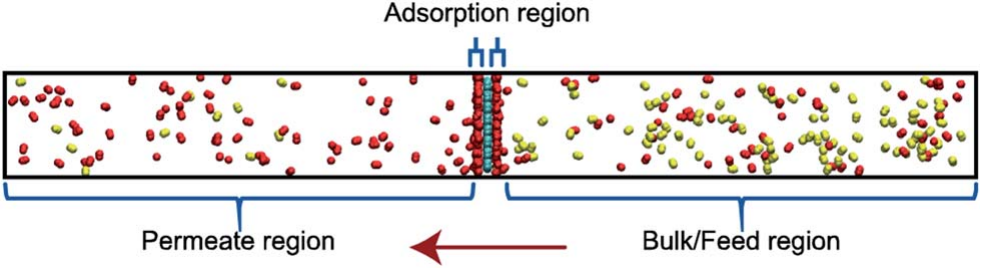
Simulation system is demarcated to three regions: bulk/feed, permeate and adsorption regions. Molecules permeate from bulk to the permeate region in the direction of the red arrow. Oxygen and nitrogen atoms are depicted in red and yellow respectively.

Classical molecular dynamics (MD) simulations were performed to examine the selectivity of O_2_ and N_2_ permeating through a nanoporous graphene (NPG) membrane using LAMMPS (Large-scale Atomic/Molecular Massively Parallel Simulator).^48^ For this study, we considered a nanoporous graphene membrane, with a bulk and permeate region each measuring 3.2 × 3.6 × 17.5 nm^3^ separated by a fixed 3.2 × 3.6 nm^2^ membrane (see Fig. 3). Fixing the membrane was found to give similar permeation behavior as allowing all but one of the membrane atoms to move, and thus for simplicity the membranes were held rigid in the simulations. NPG has been pursued as a membrane material owing to high permeabilities, mechanical strength, and chemical resilience.^41–47^ There are many computational studies using NPG membranes to investigate permeability and selectivity for gases.^35,37,41–46^ In the present work, we utilize graphene as a 2D membrane template to explore the effects of modifying interaction strengths. A nanopore of radius 6.2 Å was created in order to ensure unhindered transport of both gases. We used periodic boundary conditions in the *x* and *y* direction and reflective boundary conditions in the *z* direction. The gas pressure was calculated using the ideal gas equation of state. Initially, the feed side pressure was around 69 atm (see ESI† Section 1.5). The initial geometry consisted of 100 molecules each of N_2_ and O_2_ in the bulk region.

Each MD calculation was performed within the NVT ensemble with a Nose–Hoover thermostat at constant temperature of 500 K. A timestep of 1 fs was used for a total of 2 × 10^8^ timesteps. Each calculation was repeated with 10 different initial gas configurations. All the interactions were modelled using Lennard-Jones (LJ) potential (see ESI† Section 1.1) with the gases being modelled as rigid entities.

#### The classical force field model

To simulate the effects of increasing the adsorption energy of an O_2_ gas molecule on the NPG membrane, we tuned the LJ interaction parameter epsilon, which represents the depth of the potential well (see ESI† Section 1.1). We scaled epsilon of O atoms with the C atoms in the membrane to multiples of its original value. In this study, we consider eight values of *ε*_C–O_ (kcal mol^−1^) = 0.1, 0.2, 0.3, 0.4, 0.5, 0.6, 0.7, and 1.0, keeping all other parameters fixed. The calculation of selectivity as a function of adsorption energy difference translates to calculating selectivity as a function of the difference between the LJ interaction parameter for both the gases. To estimate the effect of changing the LJ parameter on the adsorption energy, we simulated one single O_2_ and N_2_ molecule (separately) at varying distances from the same graphene layer (without the pore) used before. For every simulation run with a specific LJ parameter, the minimum energy corresponds to the stable adsorbed state and was assigned as the adsorption energy (Section 1.3 in the ESI†).

**Fig. 4:**
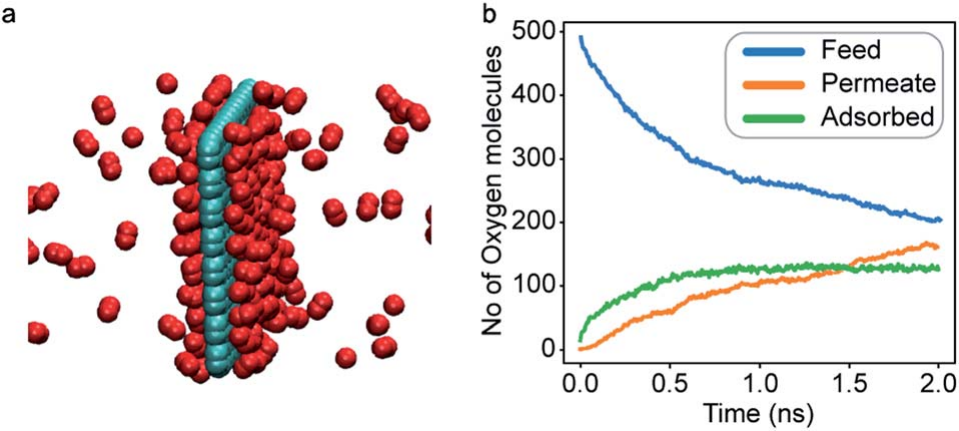
(a) A representative ‘membrane’ obtained after the pre-equilibrium run to simulate increased O_2_ adsorption. (b) Oxygen molecules in various regions of the simulation cell during a pre-equilibrium run; after some time, a saturation of O_2_ molecules in the adsorption region is obtained.

An increase in LJ interaction parameter leads to increasing O_2_ surface concentration on the membrane, as expected. Before permeation experiments were run, the surface concentration of oxygen was allowed to reach its equilibrium value by running pre-equilibrium runs. Initially, 500 O_2_ molecules were placed in the bulk and allowed to permeate and adsorb. The adsorption zone is defined as the region within 5 Å of the membrane as shown in the Fig. 3 as gas density was found to decay to its gas phase value at distances greater than 5 Å. After the saturation of O_2_ molecules in the adsorption zone (Fig. 4b), the membrane along with all the O_2_ molecules in the adsorbed zone becomes the new ‘membrane’ through which the both N_2_ and O_2_ are allowed to permeate (Fig. 4a).

#### The theoretical framework

The number of molecules in the permeate region (*N*) as a function of time (*t*) was found to follow eqn (2), where *a* and *b* are constants for a given gas molecule.^35^ (The derivation is provided in Section 1.4 in the ESI†)

2 *N* = *a*(1 − e^−*bt*^)

The form of eqn (2) is very similar to the framework used by Sun *et al.*^35^ to model the permeate. This form ensures that we start with 0 molecules in the permeate side and the number of molecules in the permeate side saturates as *t* → ∞, as one would expect in steady state conditions. The data obtained from the MD run is consequently fitted to eqn (2) to obtain the constants. Flow rate and selectivity are defined in eqn (3)–(5). Our formulation of *N* leads to modified definitions of these quantities.3
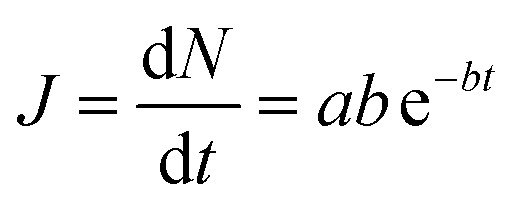
4
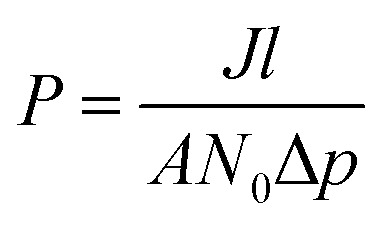
5
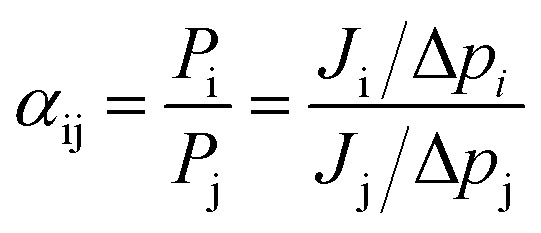
Here, *J* is the flow rate, *N* is the number of molecules in the permeate region, *t* is the time, *p* is the partial pressure, *A* is the surface area, *l* is the thickness, *N*_0_ is the Avogadro constant, *P* is the permeability, and *α* is the selectivity.

### Density functional theory

Density Functional Theory (DFT) was employed using Vienna *Ab initio* Simulation Package (VASP)^49,50^ to obtain the adsorption energies of oxygen and nitrogen molecules on two transition metal oxides: α-Fe_2_O_3_ and Co_3_O_4_ serving as candidate materials for the membrane. The factors leading to selection of these transition metal oxides are discussed in Section 4.1.† While α-Fe_2_O_3_ and Co_3_O_4_ (generally, in a composite) are currently in use in membrane applications, their use is primarily as catalysts for a reaction.^51–54^ This work explores their application as membranes solely *via* adsorption (the parameters used in the calculations are listed in Section 2 of the ESI†). Oxygen and nitrogen molecules were placed in various initial configurations and the adsorption energy (*E*_ads_) was calculated by using eqn (6).

6 *E*_ads_ = *E*_adsorbate/slab_ − (*E*_slab_ + *E*_adsorbate_)

where *E*_adsorbate/surface_ is the total energy of the adsorbate/slab system, *E*_*s*lab_ is the total energy of the substrate and *E*_adsorbate_ is the energy of the isolated molecule (O_2_ or N_2_) respectively. All the calculations were done with the same dimensions for a given system. A negative *E*_ads_ implies an exothermic process and a stable adsorption.

#### α-Fe_2_O_3_ (0001) slab

α-Fe_2_O_3_ is the most prevalent Fe(iii) oxide. It has a hexagonal close-packed structure composed of O ion with Fe ions occupying the octahedral positions. The (0001) surface was used in this study as it is the most stable at ambient conditions.^55,56^

**Fig. 5:**
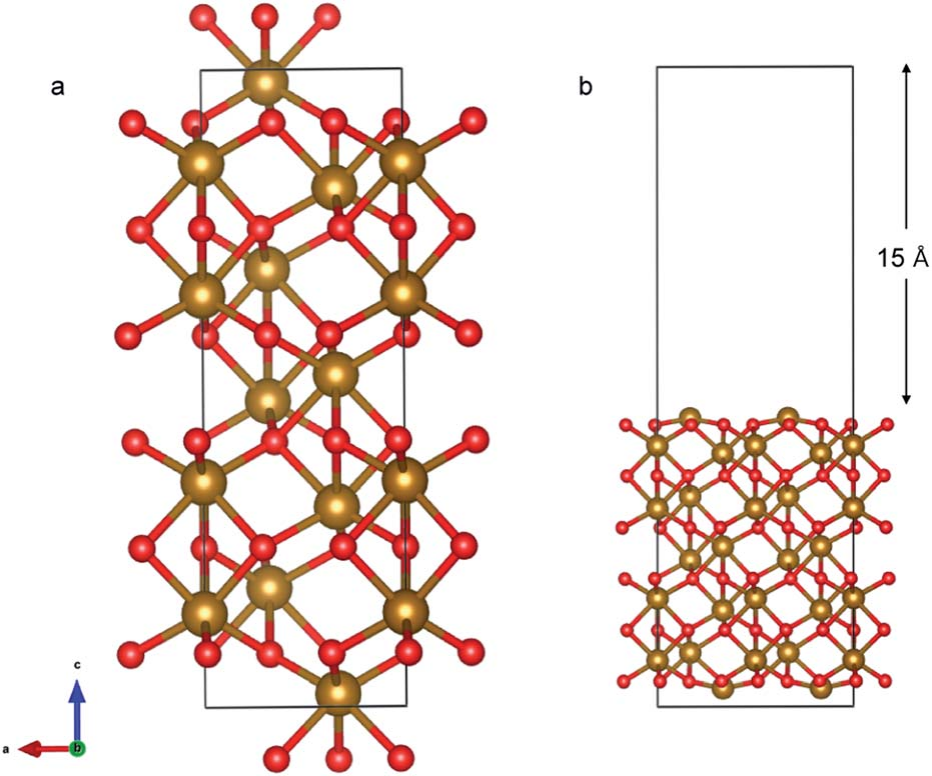
(a) Bulk Fe_2_O_3_ structure; each Fe bilayer (yellow atoms) has the same magnetic moment and is terminated by one layer consisting of 3 O atoms (red). Fe bilayers separated by the O layer have opposite magnetic moments rendering the overall structure antiferromagnetic. (b) Fe_2_O_3_ (0001) slab with Fe–O_3_–Fe termination.

The α-Fe_2_O_3_ (0001) surface consists of an iron bilayer followed by an O layer as shown in Fig. 5a. The neighboring bilayers are antiferromagnetically coupled resulting in an antiferromagnetic material.^57^ A Fe–O_3_–Fe termination as shown in Fig. 5b was used in this work. A 2 × 2 cell was used with 15 Å of vacuum in the out-of-plane direction to prevent interaction with neighboring cells. Slabs of 18 atomic layers were modeled with the middle 6 layers fixed.

#### Co_3_O_4_ (110) slab

**Fig. 6:**
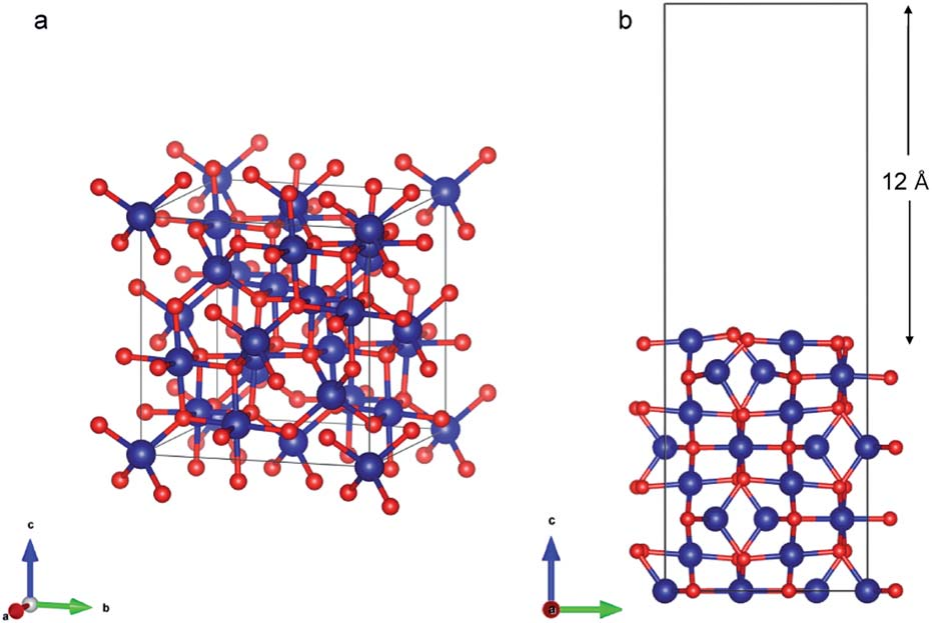
(a) Bulk Co_3_O_4_ structure (b) Co_3_O_4_ (110) slab with B termination.

The spinel Co_3_O_4_ is the thermodynamically stable cobalt oxide under ambient conditions. It has a cubic close-packed structure of O^2−^ ions where one-eighth of the tetrahedral positions are occupied by Co^2+^ ions and half of the octahedral positions are occupied by Co^3+^.^59^ The Co^2+^ ions display high magnetic spin but are coupled to ensure the overall structure is antiferromagnetic. We modeled the (110) surface (Fig. 6a) as it is one of the naturally occurring crystal faces^58^ and has high catalytic activity.^59,60^

Co_3_O_4_ (110) surface has two terminations: type A, which has two Co^2+^, two Co^3+^ and four O^2−^ ions in the surface layer, and more stable type B, which exposes two Co^3+^ and four O^2−^ ions. The more stable B termination^61^ has been modeled in this work (Fig. 6b). A 1 × 1 cell was used with 12 Å of vacuum in the out-of-plane direction to prevent interaction with neighboring cells. Slabs of 8 atomic layers were modeled with the last 3 layers fixed. Dipole corrections were added in the end.

## Results and discussion

### Selectivity

In general, results of the MD simulations showed oxygen permeating preferentially for the cases where its LJ interaction parameter is higher than that of nitrogen. Because oxygen adsorbs more strongly in those cases, desorption becomes the rate limiting factor that determines its permeability. Despite a higher energy barrier for oxygen, we still observe an increasing oxygen permeability as the LJ parameter increases. This is because of the higher concentration of oxygen in the adsorption zone. While the desorption rate depends on the energy barrier, it also depends on the concentration, and we see the effect of concentration dominating in this simulation.

**Fig. 7:**
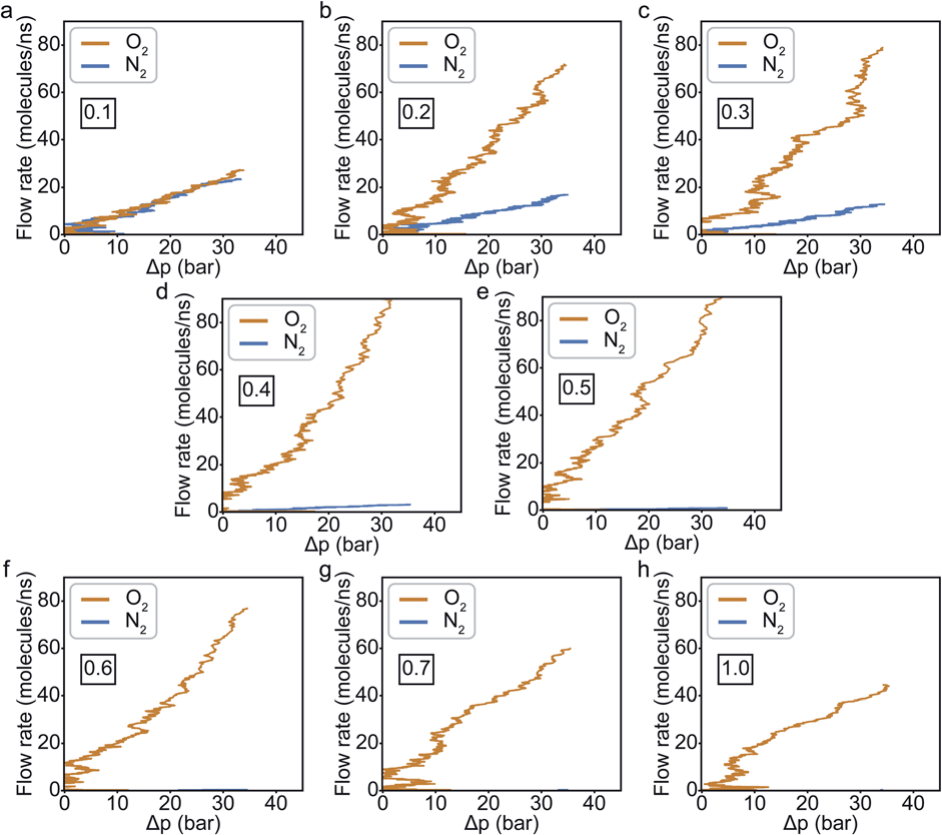
Flow rate of O_2_ and N_2_ as a function of their partial pressures at the following values of ε_C−O_ (labeled on each plot): (a) 0.1, (b) 0.2, (c) 0.3, (d) 0.4, (e) 0.5, (f) 0.6, (g) 0.7, and (h) 1.0 kcal mol^−1^. The LJ parameter for the C–N interaction is kept constant at around 0.1 kcal mol^−1^.

Fitting the number of molecules in the permeate region and consequently obtaining flow rate from eqn (3)–(5), we plot the flow rate obtained for each of the eight cases in Fig. 7. The slope of the flow rate with respect to the pressure differential is proportional to permeability (from eqn (4)) and the ratio of the slopes between the N_2_ and O_2_ give the selectivity (from eqn (5)). A 4-fold increase of the LJ parameter increases the selectivity by around 25 times.

**Fig. 8:**
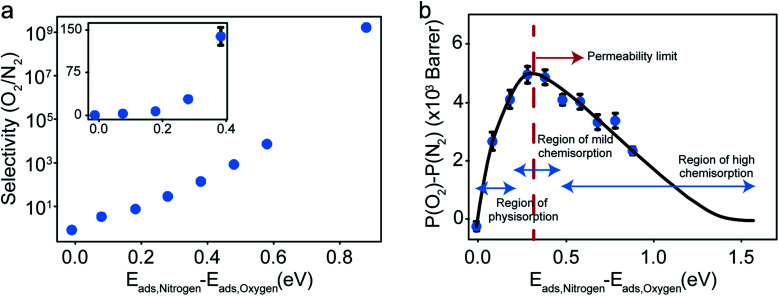
(a) Selectivity as a function of difference in adsorption energies. (b) Difference of permeabilities as a function of difference in adsorption energies and the hypothesized dependence showing the permeability limit and various chemisorption regimes of oxygen gas.

The selectivity and the difference of permeabilities as a function of adsorption energy differences corresponding to each of the eight different interaction parameters is plotted in Fig. 8a and b respectively. This trend hints at how important of a role adsorption energy can play in tuning selectivity.

Modeling efforts have traditionally used measures like selectivity, permeability and in cases of process modeling, parameters like module separation efficiency (defined as ratio of concentration in feed to concentration in retentate),^62^ and mole fractions in feed, retentate, and permeate.^63^ In this work, we have included difference of permeabilities as a useful measure to depict performance. The difference of permeabilities is useful in depicting the extent to which the decrease/increase in oxygen permeability outpaces the decrease/increase in nitrogen permeability across the entire adsorption energy difference range. We theorize that the difference of permeabilities depends on the difference of adsorption energies as shown in Fig. 8b. When neither gas is preferentially adsorbed over the other, we expect a value of 0 (indicating no selectivity of O_2_ over N_2_). As one of the gases (here, O_2_) adsorbs more strongly, the permeability difference rises initially. However, while the selectivity remains high, irreversible bonding of O_2_ molecules at very high adsorption energies results in a reduced permeance, as O_2_ would require high temperatures to desorb. Thus, these membranes are expected to have an optimal range of C–O bonding energy that leads to selectivity. This is given by the permeability limit occurring around an energy difference of 0.3 eV indicated in Fig. 8b, which corresponds to a selectivity of around 30. The permeability limit is defined as the adsorption energy difference where the difference in permeabilities of oxygen and nitrogen is maximized. Beyond the permeability limit (∼0.3 eV), the permeability of oxygen decreases along with the permeability of nitrogen. For an optimal membrane performance, both permeability and selectivity should be high. While the selectivity rises with increased adsorption energy difference, oxygen gas permeability begins to decline after this permeability limit, making the permeability limit the desired state of functioning if the process is optimized for maximum permeability. In this work, we observe that the permeability limit also corresponds to the adsorption energy difference that leads to maximized oxygen permeability. However, if the maxima were to occur at two different points, the difference of permeabilities approach could help in optimizing processes subject to maximizing oxygen permeability and selectivity while simultaneously minimizing nitrogen permeability. Predicting this limit quantitatively is non-trivial as it would require exhaustive lists of adsorption energies of O_2_ and N_2_ in various configurations and multiple adsorption modes (like molecular and dissociative adsorption), although it can be estimated using desorption temperatures (*T*_des_).^64,65^ In order to maximize selectivity at ambient temperatures, they should be optimized to a binding energy difference corresponding to the permeability limit. This can be achieved by ensuring the maximum desorption (and hence permeability) of O_2_ gas (or the more preferentially adsorbed species) occurs at ambient temperatures, or in other words, the desorption temperature lies close to ambient temperatures. Thus, the search for materials with adsorption energy differences that are close to the permeability limit translates to materials with desorption temperature close to ambient temperatures.

Our strategy assumes only those modes of adsorption are active which correspond to the ones that have desorption temperature close to ambient temperature. Usually, materials have multiple modes of oxygen adsorption available at any given temperature, like physisorption, chemisorption, or dissociative chemisorption on a specific site. While we cannot prevent other modes of adsorption from occurring, the strategy discussed here can still be adapted in those scenarios with some modifications. The adsorbates on sites that opt for another mode of adsorption (like dissociative adsorption that occurs in the strong chemisorption regime) will either demonstrate a desorption temperature higher or lower than the ambient temperatures. The former will correspond to a case where the entities are irreversibly bound making the corresponding site inactive. The latter would still result in permeability. These can potentially impact the selectivity obtained. One way to reduce this impact would be to choose a material with limited adsorption modes (the ones with desorption around ambient temperatures). There are some surfaces like Pt (111) that allow only for one mode of O_2_ chemisorption, but their desorption temperatures are in the range of 800–925 K.^66^ For achieving limited adsorption modes with desorption temperatures at ambient temperatures, methods like co-adsorption, introducing strain, and alloying may need to be employed to alter adsorption modes.

Transition metal oxides often have large differences between their oxygen and nitrogen adsorption energies without binding the oxygen too strongly, making them good initial candidates for a membrane with an optimal oxygen permeability.^64,65,67^ Adsorption of molecular adsorbates on transition metal systems offer a good range of adsorption energies, with the strength of the chemisorption bond related to the interaction of the adsorbate with metal *s* and d bands. Chemisorption becomes stronger when the interaction with the metal surface gives rise to emptier anti-bonding states, a direct result of d bands shifting up relative to the Fermi energy. Hence the chemisorption energy tends to rise from right to left across a period in the periodic table, and decreases down a column because of the increasing repulsion due to the Pauli principle.^68^

Transition metal systems provide a good set of elements to choose from that ensure we remain in the mild chemisorption regime that is crucial for the functioning of the membrane. In order to ensure significant oxygen permeability at ambient temperatures, we require ease in oxygen desorption at those temperatures. If the modes of oxygen adsorption in some materials were such that their corresponding desorption temperatures were around ambient temperatures, those materials would be ideal to pursue as candidates for adsorption-based membranes. Some modes of adsorption in Fe_2_O_3_ and Co_3_O_4_ result in a desorption temperature around 300 K (ref. 67) and thus, we focus on them as examples.

To model these transition metal oxides as membranes for O_2_ and N_2_ separation, we require forcefields that can successfully simulate the chemisorption between O_2_ molecules and the transition metal oxide surfaces. Reactive forcefields (ReaxFF) are well suited for this task, but there are no suitable ReaxFF parameterizations for these systems, making a full-fledged permeation simulation using MD challenging. Thus, to gauge the suitability of these materials, we instead carry out a preliminary investigation to determine the adsorption energies of O_2_ and N_2_ on some oxide surfaces. Using this preliminary examination, we can verify if the material properties lie in the range desired, which could in turn motivate future work on more extensive permeation simulations.

### Fe_2_O_3_ (0001) Fe–O_3_–Fe termination

The optimized bulk and surface properties are listed in Table S2.† A good agreement with previous experimental as well as computational work is obtained.^57,69–77^ We performed adsorption calculations on the relaxed structure obtained. While many adatom adsorption configurations exist, only a handful of them are stable. Thus, the final configurations obtained by previous DFT studies^76,77^ were used as the initial configurations of O_2_ and N_2_ molecules in this study.

**Fig. 9:**
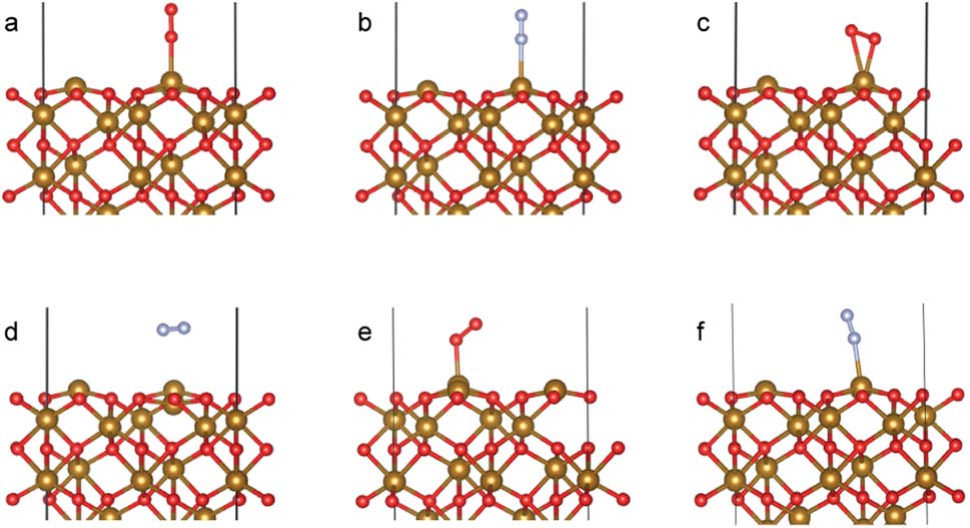
Adsorption configurations for O_2_ (red atoms) and N_2_ (grey atoms) on Fe_2_O_3_ (0001) surface with Fe–O_3_–Fe termination: (a) O_2_ perpendicular*, (b) N_2_ perpendicular, (c) O_2_ parallel, (d) N_2_ parallel*, (e) O_2_ oblique and (f) N_2_ oblique. * refers to calculation where the in-plane positions of the gas molecules are fixed.

An O_2_ molecule was placed in 3 different configurations, perpendicular, parallel, and oblique to the surface. The DFT calculations find the oblique configuration to be the most energetically stable, in agreement with the previous calculations.^76^ In contrast to O_2_, the N_2_ molecule was found to flip into a perpendicular position even when the initial geometry was a parallel or oblique configuration, also in agreement with previous calculations.^77^ While our relaxed geometries closely resemble the ones obtained in previous studies, the adsorption energies vary due to the use of different functionals and other parameters. The adsorption energy along with the geometry parameters of the relaxed structure for both the molecules are presented in Table 1. The relaxed structures are depicted in Fig. 9.

**Table 1:** The adsorption energies and configuration parameters for O_2_ and N_2_ on Fe_2_O_3_ (0001) surface with Fe–O_3_–Fe termination. * refers to calculations where the in-plane positions of the gas molecules were fixed

	Perpendicular			Parallel			Oblique			
	[pw]		[57]	[pw]		[57]	[pw]		[76]	[57]
	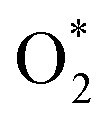	N_2_	O_2_	O_2_	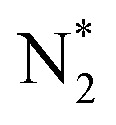	O_2_	O_2_	N_2_	O_2_	O_2_
*E*_ads_ (eV)	−0.17	−0.36	−0.7	−0.3	−0.09	−1.0	−0.45	−0.36	−0.87	−0.5
Fe–X (Å)	2.05	2.25	1.8	2.04	3.54	1.9	2.08	2.24	2.07	2.7
X–X (Å)	1.25	1.11	1.27	1.29	1.11	1.34	1.26	1.11	1.53	1.25

A difference between the adsorption energies of O_2_ and N_2_ of about 0.1 eV is obtained, which can provide a selectivity of around 3 when used in the same conditions as our MD model. O_2_ was found to chemisorb while it is a purely physisorption process for N_2_. The adsorption energy of O_2_ is not large enough to lie in the strong chemisorption regime, ensuring any O_2_ adsorbed is free to desorb and eventually permeate through the membrane.

### Co_3_O_4_ (110) B termination

As with the case of Fe_2_O_3_, optimized bulk and surface properties are listed in Table S3† and good agreement with previous experimental as well as computational work is obtained with our DFT calculations.^66,78–84^ Adsorption calculations were again performed on the relaxed structures.

**Fig. 10:**
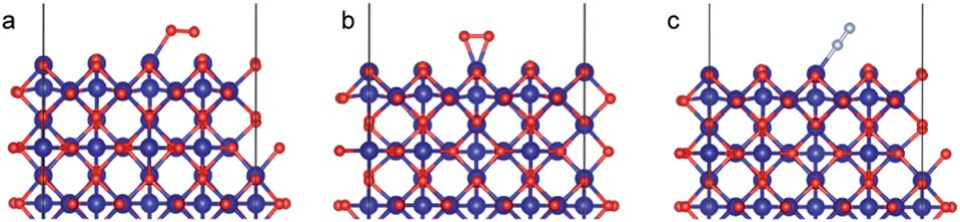
Adsorption configurations for O_2_ (red atoms) and N_2_ (grey atoms) on Co_3_O_4_ (110) surface with B termination: (a) O_2_ parallel-1, (b) O_2_ parallel-2 and (c) N_2_ perpendicular.

Two initial configurations for the O_2_ molecule were considered based on the relaxed configurations obtained by Xu *et al.*^83^ and Wang *et al.*^84^ In the first geometry, O_2_ was placed parallel to the surface and on top of a Co^3+^ ion (termed parallel-2) while in the second one, O_2_ is placed at an oblique angle in the bridge site between a Co^3+^ ion in the surface and a Co^2+^ ion of the next layer (termed parallel-1). The latter relaxed to a configuration with the molecules parallel to the surface and displaced with respect to the parallel-2 configuration (Fig. 10b). The parallel-1 configuration was found to be more stable than parallel-2. While other studies have also concluded parallel-1 as the lowest energy configuration, our parallel-1 geometry was slightly different. The adsorption energies were strikingly similar, despite differences in functionals and other parameters used. The N_2_ molecule was placed perpendicular to the surface and on top of a Co^3+^ ion. We found the Co_3_O_4_ system very sensitive to the magnetic moments and found that it was important to use the magnetic moments obtained for the relaxed surface as the initial magnetic moments for the next set of geometry relaxations. The adsorption energy along with the geometry parameters of the relaxed structure for both the molecules are presented in Table 2. The relaxed structures are shown in Fig. 10.

**Table 2:** The adsorption energies and configuration parameters for O_2_ and N_2_ on Co_3_O_4_ (110) surface with B termination

	[pw]			[84]	[83]
	Parallel-1	Parallel-2	Perpendicular	Parallel-1	Parallel-1
	O_2_	O_2_	N_2_	O_2_	O_2_
*E*_ads_ (eV)	−1.20	−0.92	−1.00	−1.20	−1.18
Co–X (Å)	2.03	1.93	1.95	1.88	1.88
X–X (Å)	1.25	1.30	1.11	1.33	1.30

**Fig. 11:**
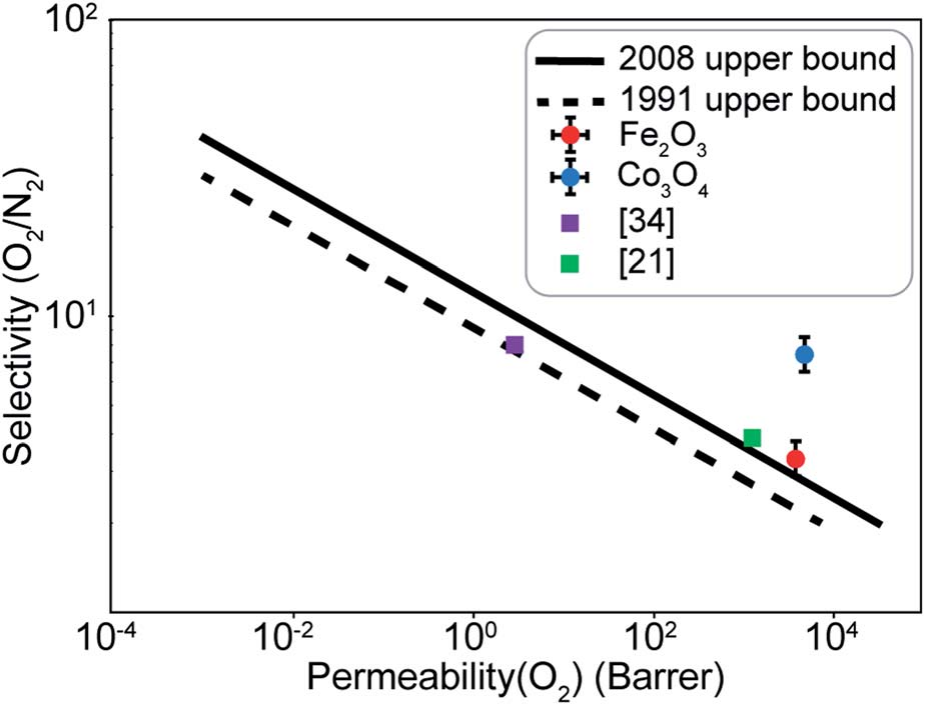
Selectivity and permeability for the Fe_2_O_3_ and Co_3_O_4_ membranes compared to the present (2008), prior (1991) Robeson upper bounds,^8^ and experimental data from ref. 21 and 34. Upper bounds adapted from ref. 8.

A difference of about 0.2 eV is obtained between the O_2_ and N_2_ adsorption energies, which can provide a selectivity of around 8 when used in the same conditions as our MD model. Similar to the Fe_2_O_3_ system, O_2_ was found to chemisorb while it is a purely physisorption process for N_2_. Co_3_O_4_, just like Fe_2_O_3_, has O_2_ adsorption energy in the mild chemisorption regime ensuring that this membrane can also potentially function within the permeability limit. These results show that there exist materials with properties lying in the desired range of adsorption energies. Combining with the permeability and selectivity trend obtained using MD calculations allows us to connect these DFT adsorption calculations to theoretical selectivity performance of different material systems. By placing these materials in a system where adsorption, rather than simple translocation, is the main mechanism of permeation through the membrane, we predict selectivity between N_2_ and O_2_ from 3–8, achieved by differences in the adsorption energies of the species on the membrane rather than membrane pore sizes, provided the pore size is large enough to allow unhindered permeation of all species. Fig. 11 depicts the selectivity *vs.* permeability for each transition metal oxide membrane along with the present (2008) and prior (1991) Robeson upper bounds^8^ along with some experimental data for comparison. We observe that they lie above the bounds and hence, these membranes have the potential to perform better than the polymer membranes. We want to highlight that the selectivity and permeability of the metal oxide membranes are a rough estimate, and are subject to the approximation that permeation characteristics in metal oxide membranes are similar to NPG, which may not be true. Future work to obtain the permeability and selectivity is required to substantiate these claims. The objective of our study was to shed light on the effect of adsorption energy differences on the selectivity and permeability, combined with DFT calculations to support the suitability of materials that meet the given criteria for permeability and selectivity.

There are additional important parameters like pore geometry, density, and functionalization that have not been considered in this work and should be considered in a future study. Previous studies^38–40^ on surface flow membranes that use a similar strategy of competitive adsorption have suggested that the pore diameter should be larger than the diameter of the adsorbing species, but smaller than four times the diameter of the largest gas molecule. The latter condition prevents the fast diffusion of the less favourably adsorbed species. Though synthesis of membranes meeting this criterion is a separate challenge, previous work^38–40^ has already demonstrated the creation of membranes from 2D materials that can meet the necessary pore-size criteria. It is also reasonable to believe that membranes with appropriate pore sizes could be created through particle sintering^85^ or other techniques.^86^ Using SSF™ nanoporous carbon membranes, Rao and Sircar^39^ demonstrate that pore diameters should typically lie between 4–15 Å for optimal permeability. Moreover, for N_2_ at pore diameters higher than 1000 Å, bulk diffusion is dominant while permeation is predominantly *via* Knudsen diffusion if the pore diameters lie between 10–1000 Å. Since these membranes provide optimal selectivity when the adsorbed-phase pathway is activated for the gases, the pore sizes should lie in the regime corresponding to activated diffusion and pore diameters higher than 10 Å may lead to lowered selectivity.

Moreover, while in this study, we have assumed the high permeabilities obtained from MD simulation of a thin NPG membrane to serve as a reasonable proxy for a potential metal oxide membrane, we want to emphasize that the permeabilities can potentially change with increased membrane thickness. However, we believe that O_2_ permeation might be unaffected by the thickness to a large degree. This is because desorption is the rate limiting step in O_2_ permeation. Oxygen permeation is a strictly permeate side phenomena and independent of the oxygen diffusion through the pores. As long as the thickness is such that the rate determining step does not shift from desorption to diffusion, it has minimal impact on oxygen permeation. In the case of N_2_, increasing membrane thickness can impact its permeability to a larger degree because, for N_2_, diffusion can become its rate limiting step when the concentration of oxygen in the adsorption region becomes substantial. However, if we can achieve a pore geometry such that the membrane surface exposed within the pore behaves similar to the outer membrane surface, then we can consider the membrane area exposed within the pore as an extension of the external surface and assume that it would display similar adsorption/desorption properties as that of the surface. Since the thickness of the membrane determines permeance, for acceptable permeance, Rao and Sircar^39^ recommend that membrane thickness should be less than 5 μm.

Another caveat to consider is the activation energy barrier for association and dissociation *via* surface diffusion as depicted by energy barriers 2 and 4 in Fig. 1b. These energy barriers are a function of the membrane surface heterogeneity and thus metal oxides may have a higher barrier than NPG membranes. Generally, the activation energy for diffusion is smaller than desorption energy.^87^ However, even in the limiting case where the diffusion energy barrier is higher than the desorption energy barrier, surface transport of gas would switch to a predominantly adsorption–desorption mechanism, resulting in desorption still being the rate determining step. Regardless of whether the diffusion barrier becomes higher than the desorption barrier, since metal oxides have higher diffusion energy barriers compared to NPG, we expect the overall flux and hence the gas permeability to decrease. In other words, permeability is maximized if the other energy barriers are much lower than the desorption energy barrier. Methods like co-adsorption, introducing strain, and alloying may prove useful in engineering the surface to minimize the diffusion barrier.^68^ While adsorption energy difference is a primary factor determining selectivity and permeability in these membranes, future work to investigate the effect of other parameters is important. Moreover, we want to emphasize that our work was aimed at studying the range of adsorption energy differences most suited for O_2_/N_2_ separation with further DFT calculations to preliminarily prove that materials that lie in the desired range exist. Future work to determine the permeability and selectivity of such metal oxide membranes is essential to determine its applicability.

A better selectivity as well as permeability can be obtained from materials that provide a difference in adsorption energies of around 0.3 eV. Other materials such as MnO_2_ and NiO are worth exploring towards this end, as well as an examination of the ability to synthesize these materials in the required configurations. This approach to gas separation could potentially be generalized to the separation of wide range of gas species, where the desired gas permeate displays mild chemisorption and the others prefer physisorption.

## Conclusions

In this work, we highlight the importance of adsorption in the separation processes of species when size-based sieving is not a viable option, such as in air separation. In particular, we used computational approaches to demonstrate the potential for increased selectivity of O_2_ over N_2_ by changing the energy profile of the adsorption-based pathways using DFT calculations and MD simulations. First, we showed that a process where desorption serves as the rate limiting process can lead to increased selectivity in a nanoporous membrane. We also explored the limits of such a procedure and discussed what constraints a membrane material ought to satisfy to be viable for this approach. Lastly, we demonstrated using DFT calculations how the binding energies of two transition metal oxides, Fe_2_O_3_ and Co_3_O_4_, fulfil the discussed energy requirements, being neither too weak to prevent competition between oxygen and nitrogen nor too strong to prevent oxygen desorption. While there might be additional conditions that will emerge as we examine other nuances of material properties, these adsorption energies provide a suitable motivation to consider adsorption-based pore-flow membranes as a viable option for air separation.

## Author contributions

The work was conceived and manuscript written through contributions of all authors.

## Conflicts of interest

There are no conflicts to declare.

## Supplementary Material

NA-003-D1NA00307K-s001
