# Supplementary material for: Adsorption-based membranes for air separation using transition metal oxides
Source: Nanoscale Adv. 2021 Jun 25;3(15):4502–12. doi: 10.1039/d1na00307k (PMC9418459; doi:10.1039/d1na00307k)
Supplement: NA-003-D1NA00307K-s001 [file NA-003-D1NA00307K-s001.pdf]

Electronic Supplementary Material (ESI) for Nanoscale Advances.
This journal is © The Royal Society of Chemistry 2021

Supporting information

Adsorption-based membranes for air separation using metal oxide membranes

Asmita Jana, David S. Bergsman, and Jeffrey C. Grossman

1. Molecular Dynamics simulations

1.1 Lennard-Jones potential

In this work, all the interactions were modeled using the Lennard-Jones (LJ) potential with LAMMPS.[1] A description of this potential is found in [2]. The form of the LJ 12-6 potential used is given by

$$E(r) = \begin{cases} 4\varepsilon\left[\left(\frac{\sigma}{r}\right)^{12} - \left(\frac{\sigma}{r}\right)^{6}\right] & (r < r_{cut}) \\ 0 & (r \geq r_{cut}) \end{cases} \qquad (S1)$$

A cutoff radius (r_{cut}) of $10\ \text{Å}$ was used. The values of σ and ε used in the simulations are listed in table S0.

Table S0: LJ potential parameters used in the simulation

	ε (kcal/mol)	σ (Å)
C-N	0.102	3.516
C-O	0.1, 0.2, 0.3, 0.4, 0.5, 0.6, 0.7, 1.0	3.401
N-N	0.077	3.263
N-O	0.086	3.148
O-O	0.096	3.033

1.1.2 Bond description

Along with the bonds in the nanoporous graphene (NPG) membrane, the bonds in the diatomic gases were also fixed in length and not allowed to vibrate.

1.2 The pore in NPG membrane

Since the selectivity isn't based on molecular sieving, we employed an NPG membrane with a pore large enough to allow unhindered motion of both gases. In the energy profile, this would ensure that the translocation step in the adsorbed-phase pathway wouldn't have the highest energy barrier.

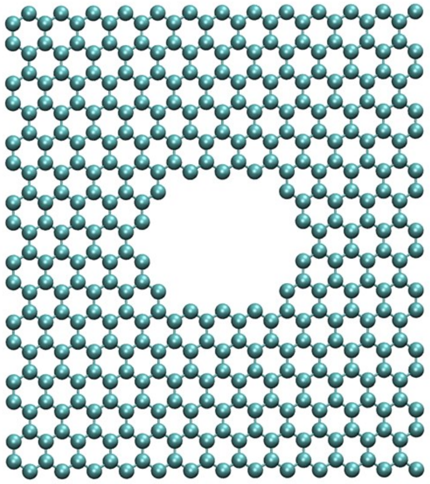

Fig S1: The bare nanoporous graphene (NPG) membrane.

1.3 Obtaining adsorption energies from LJ parameters and resulting permeability and selectivity

A setup as shown in Fig S2a was used where a dimer of O_2 and N_2 were separately placed vertically at various distances from the membrane surface and the potential energy was tabulated. The potential energy plots are depicted in Fig S2b and the minimum energies obtained for each of the LJ parameter are indicated in Table S1. The selectivity as a function of the LJ parameters is plotted in Fig S3.

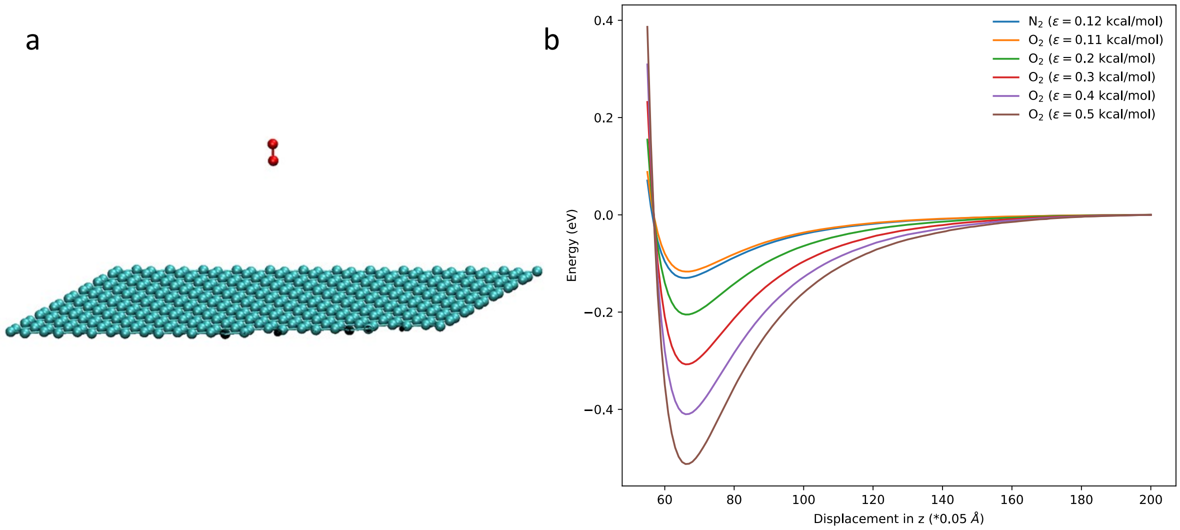

Fig S2: a. Cross section of the system used to calculate adsorption energy. b. Plot of energy vs displacement for various LJ parameters.

Permeability was calculated using equation S2

$$P = \frac{Jl}{AN_0 \Delta p} \qquad \text{(S2)}$$

Here, J is the flow rate, p is the partial pressure, A is the surface area, *l* is the thickness, N_0 is the Avogadro constant, and P is the permeability. The following values were used to compute permeability: $A = 3.6 \times 3.15 \times 10^{-18}$ m^2, $N_0 = 6.023 \times 10^{23}$, $l = 3.4 \times 10^{-10}$ m. $\frac{J}{\Delta p}$ values were obtained from the slope of the curves depicted in Figure 7.

Table S1: Adsorption energies corresponding to the LJ parameters used and the calculated selectivity

ε (kcal/mol)		E_{ads} (eV)		$E_{ads}(O_2)$-$E_{ads}(N_2)$ (eV)	Permeability (Barrer)		Selectivity (O_2/ N_2)
O_2	N_2	O_2	N_2		O_2	N_2	
0.11	0.12	-0.12	-0.13	0.01	1094±80	1335±77	0.8±0.1
0.2	0.12	-0.21	-0.13	-0.08	3695±250	1106±71	3.3±0.4
0.3	0.12	-0.31	-0.13	-0.18	4594±281	610±36	7.5±0.9
0.4	0.12	-0.41	-0.13	-0.28	4976±286	174±8	29±3
0.5	0.12	-0.51	-0.13	-0.38	4746±258	34±2	138±16
0.6	0.12	-0.62	-0.13	-0.49	3973±187	4.9±0.2	840±81

0.7	0.12	-0.72	-0.13	-0.59	3905±238	0.5±0.1	7284±1693
1.0	0.12	-1.03	-0.13	-0.9	2273±122	1E-6±4E-14	1.65E9±8E7

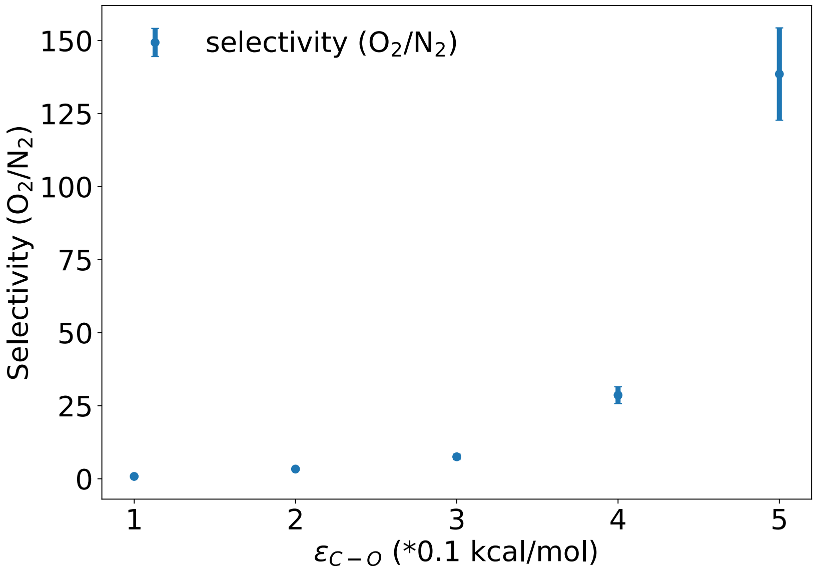

Fig S3: A plot of selectivity vs C-O LJ interaction parameter. A similar trend is observed when plotted against difference in adsorption energies.

1.4 Theoretical framework for permeability and selectivity

The theoretical framework used to obtain selectivity is by adopting the methodology used by Sun et al.[19] Flow rate expressed as a function of permeability is given by Equation S3

$$J = \frac{dN}{dt} = PA\Delta p \qquad (S3)$$

Where J is flow rate, A is the surface area, Δp is the difference of partial pressure and P is the permeability. Δp, in turn, can be expressed as a function of the initial pressure p_{in}, number of molecules in the permeate region N, number of molecules adsorbed N_{ads}, and total number of molecules N_{tot} and is given by Equation S4.

$$\Delta p = \frac{N_{tot} - N_{ads} - 2N}{N_{tot}} p_{in} \qquad (S4)$$

Combining equation S3 and S4 gives us equation S5,

$$J = \frac{dN}{dt} = PA(N_{tot} - N_{ads} - 2N)\frac{p_{in}}{N_{tot}} \qquad (S5)$$

Integrating equation S5, we get the following:

$$N = \left(\frac{N_{tot} - N_{ads}}{2}\right)\left[1 - e^{\frac{-2PAp_{in}}{N_{tot}}}\right] \qquad (S6)$$

Equation S6 is in the following form and all our calculations are fitted to the equation S7.

$$N = a(1 - e^{-bt}) \qquad (S7)$$

1.5 Pressure calculation

Pressure is calculated using the ideal gas law as shown in equation S8.

$$p = \frac{Nk_BT}{V} \qquad (S8)$$

Where p, N, k_B, T, and V represent the pressure, number of molecules, the Boltzmann constant, temperature, and volume respectively. For obtaining the initial pressure in the feed side, we use N = 200 molecules (100 O_2 and 100 N_2), T = 500 K, and V = 31.5*36*175*10^{-30} m^3. This results in an initial pressure of around 68.6 atm in the feed side.

2. Density Functional theory calculations

Density Functional Theory (DFT) was employed using Vienna Ab initio Simulation Package (VASP)[20-22] to obtain the adsorption energies of oxygen and nitrogen molecules on two transition metal oxides: α-Fe_2O_3 and Co_3O_4. The projector augmented wave (PAW) method[21] was used and the exchange-correlation effects are described by the generalized gradient approximation (GGA) as developed by Perdew, Burke and Ernzerhof (PBE).[24] Since these materials are magnetic, spin polarized calculations were performed. In order to capture the physisorption of N_2 molecules, van der Waals (vdW) forces were incorporated using the D3 correction method of Grimme et al.[25] We used an energy cutoff of 650 eV for both systems. Monkhorst-Pack *k*-points meshes of 4x4x2 (4x4x1) and 2x2x2 (2x3x1) were used for bulk (surface) Fe_2O_3 and Co_3O_4 systems, respectively. To account for correlations in the 3d orbitals in Fe_2O_3 and Co_3O_4, we used Hubbard U-J=4[26] and U-J=3[27] parameters respectively in the Dudarev approach.[28]

All the geometry relaxation was converged to within 1x10^{-5} eV of the total energy. For surface relaxations, it was found to be advantageous to relax the structure in stages. A rough relaxation to 1x10^{-4} eV of the total electronic energy and 0.03 eV/Å of total force was followed by a refined relaxation to 1x10^{-6} eV of the total electronic energy and 0.001 eV/ Å of total force. For asymmetric surfaces, a dipole correction[29,30] was added at the end.

2.1 Fe_2O_3

A lot of theoretical study have been done for the (0001) surface.[6,7] The bulk and surface properties as well as the parameters used in this study have been listed in Table S2. The lattice

parameters match closely with other theoretical studies as well as experimental ones. The surface energy obtained for the Fe-O_3-Fe termination is within the range of previous DFT studies (1.01-1.70 J/m^2)[5,7,8]

Table S2: Lattice parameters, magnetic moment and surface energy of the Fe_2O_3 system

	Present work	Berger mayer et al.[3]	Gattinoni et al.[5]	Tang and Liu[4]	Dzade et al.[11]	Wang et al.[7]	Exp.
Functional	PBE+U+D3	PBE	optB86b-vdW+U	PBE+U	PW91+U +D2	FP-LAPW	-
Energy cutoff (eV)	650	400	550	400	400	18 Ry	-
K-points mesh (bulk)	4x4x2	4x4x1	4x4x2	5x5x2	11x11x7		-
K-points mesh (surface)	4x4x1	4x4x1	4x4x1	5x5x1	5x5x1		-
U, J	5, 1	-	U-J=4	5, 1	5, 1	-	-
a (Å)	5.052	4.995	5.035	5.027	5.024	5.025	5.035[7]
c (Å)	13.823	13.858	13.763	13.728	13.658	13.671	13.747[7]
Bulk magnetic moment (μ_B)	4.159	3.5	4.24	4.15	4.23	3.39	4.6-4.9[9,10]
Surface energy (J/m^2)	1.34	-	1.54	1.28	1.66	1.52	

2.2 Co_3O_4

The bulk and surface properties as well as the parameters used in this study have been listed in Table S3. The lattice parameters match closely with other theoretical studies as well as experimental ones.

Table S3: Lattice parameters, magnetic moment and formation energy of the Co_3O_4 system

	Present work	Xu et al.[18]	Wang et al.[17]	Beatty et al.[15]	Ren et al.[14]	Dong et al.[16]	Exp.
Functional	PBE+U+D3	PBE+U	PBE+U	PBE+U+D3	PBE+U+D3	PBE+U+D3	-
Energy cutoff (eV)	650	4.5 Å	380	500	500	400	-
K-points mesh (bulk)	2x2x2	5x5x5	4x4x4	6x6x6	8x8x8		-

K-points mesh (surface)	2x3x1	2x3x1	3x2x1		2x2x1	2x2x1	-
U, J	4, 1	3, 1	4, 1	U =5.9	U-J=3	U-J=3	-
a (Å)	8.096	8.084	8.243	8.113	8.072	7.99	8.084[12], 8.065[13]
Bulk magnetic moment (μ_B)	2.608	2.631			2.26		
Formation energy (J/m^2)	-9.394	-10.435					

3. References

1. http://lammps.sandia.gov, Sandia National Laboratories
2. Mayo, S. L., Olafson, B. D., & Goddard, W. A. (1990). DREIDING: a generic force field for molecular simulations. *Journal of Physical chemistry*, *94*(26), 8897-8909.
3. Bergermayer, W., Schweiger, H., & Wimmer, E. (2004). Ab initio thermodynamics of oxide surfaces: O 2 on Fe 2 O 3 (0001). *Physical Review B*, *69*(19), 195409.
4. Tang, J. J., & Liu, B. (2016). Reactivity of the Fe2O3 (0001) surface for methane oxidation: A GGA+ U study. *The Journal of Physical Chemistry C*, *120*(12), 6642-6650.
5. Gattinoni, C., Ewen, J. P., & Dini, D. (2018). Adsorption of surfactants on α-Fe2O3 (0001): a density functional theory study. *The Journal of Physical Chemistry C*, *122*(36), 20817-20826.
6. Trainor, T. P., Chaka, A. M., Eng, P. J., Newville, M., Waychunas, G. A., Catalano, J. G., & Brown Jr, G. E. (2004). Structure and reactivity of the hydrated hematite (0 0 0 1) surface. *Surface Science*, *573*(2), 204-224.
7. Wang, X. G., Weiss, W., Shaikhutdinov, S. K., Ritter, M., Petersen, M., Wagner, F., ... & Scheffler, M. (1998). The hematite (α-Fe 2 O 3)(0001) surface: evidence for domains of distinct chemistry. *Physical Review Letters*, *81*(5), 1038.
8. Guo, H., & Barnard, A. S. (2011). Thermodynamic modelling of nanomorphologies of hematite and goethite. *Journal of Materials Chemistry*, *21*(31), 11566-11577.
9. Coey, J. M. D., & Sawatzky, G. A. (1971). A study of hyperfine interactions in the system (Fe1-xRhx) 2O3 using the Mossbauer effect (Bonding parameters). *Journal of Physics C: Solid State Physics*, *4*(15), 2386.
10. Kren, E., Szabo, P., & Konczos, G. (1965). Neutron diffraction studies on the (1-x) Fe_2O_3 – x Rh_2O_3 system. *Phys. Letters*, *19*
11. Dzade, N. Y., Roldan, A., & De Leeuw, N. H. (2014). A density functional theory study of the adsorption of benzene on hematite (α-Fe2O3) surfaces. *Minerals*, *4*(1), 89-115.
12. Smith, W. L., & Hobson, A. D. (1973). The structure of cobalt oxide, Co3O4. *Acta Crystallographica Section B: Structural Crystallography and Crystal Chemistry*, *29*(2), 362-363.
13. Hu, L., Peng, Q., & Li, Y. (2008). Selective synthesis of Co3O4 nanocrystal with different shape and crystal plane effect on catalytic property for methane combustion. *Journal of the American Chemical Society*, *130*(48), 16136-16137.
14. Ren, J., Song, K. H., Li, Z., Wang, Q., Li, J., Wang, Y., ... & Kim, C. K. (2018). Activation of formyl CH and hydroxyl OH bonds in HMF by the CuO (1 1 1) and Co3O4 (1 1 0) surfaces: A DFT study. *Applied Surface Science*, *456*, 174-183.
15. Beatty, J., Cheng, T., Cao, Y., Driver, M. S., Goddard III, W. A., & Kelber, J. A. (2017). Nucleation of graphene layers on magnetic oxides: Co3O4 (111) and Cr2O3 (0001) from theory and experiment. *The Journal of Physical Chemistry Letters*, *8*(1), 188-192.

16. Dong, H., Zheng, Y., & Hu, P. (2019). A DFT study of direct furfural conversion to 2-methylfuran on the Ru/Co 3 O 4 surface. *Physical Chemistry Chemical Physics*, *21*(3), 1597-1605.
17. Wang, Z., Liu, J., Yang, Y., Liu, F., Yu, Y., & Yan, X. (2020). Molecular Mechanistic Nature of Elemental Mercury Oxidation by Surface Oxygens over the Co3O4 Catalyst. *The Journal of Physical Chemistry C*, *124*(8), 4605-4612.
18. Xu, X. L., Chen, Z. H., Li, Y., Chen, W. K., & Li, J. Q. (2009). Bulk and surface properties of spinel Co3O4 by density functional calculations. *Surface Science*, *603*(4), 653-658.
19. Sun, C., Boutilier, M. S., Au, H., Poesio, P., Bai, B., Karnik, R., & Hadjiconstantinou, N. G. (2014). Mechanisms of molecular permeation through nanoporous graphene
20. Kresse, G., & Hafner, J. (1993). Ab initio molecular dynamics for liquid metals. *Physical Review B*, *47*(1), 558.
21. Kresse, G., & Furthmüller, J. (1996). Software VASP, vienna (1999). *Phys. Rev. B*, *54*(11), 169.
22. Kresse, G., & Furthmüller, J. (1996). Efficiency of ab-initio total energy calculations for metals and semiconductors using a plane-wave basis set. *Computational materials science*, *6*(1), 15-50.
23. Kresse, G., & Joubert, D. (1999). From ultrasoft pseudopotentials to the projector augmented-wave method. *Physical review b*, *59*(3), 1758.
24. Perdew, J. P., Burke, K., & Ernzerhof, M. (1996). Generalized gradient approximation made simple. *Physical review letters*, *77*(18), 3865.
25. Grimme, S., Antony, J., Ehrlich, S., & Krieg, H. (2010). A consistent and accurate ab initio parametrization of density functional dispersion correction (DFT-D) for the 94 elements H-Pu. *The Journal of chemical physics*, *132*(15), 154104.
26. Huang, X., Ramadugu, S. K., & Mason, S. E. (2016). Surface-specific DFT+ U approach applied to α-Fe2O3 (0001). *The Journal of Physical Chemistry C*, *120*(9), 4919-4930.
27. Selcuk, S., & Selloni, A. (2015). DFT+ U study of the surface structure and stability of Co3O4 (110): Dependence on U. *The Journal of Physical Chemistry C*, *119*(18), 9973-9979.
28. Dudarev, S. L., Botton, G. A., Savrasov, S. Y., Humphreys, C. J., & Sutton, A. P. (1998). Electron-energy-loss spectra and the structural stability of nickel oxide: An LSDA+ U study. *Physical Review B*, *57*(3), 1505.
29. Neugebauer, J., & Scheffler, M. (1992). Adsorbate-substrate and adsorbate-adsorbate interactions of Na and K adlayers on Al (111). *Physical Review B*, *46*(24), 16067.
30. Makov, G., & Payne, M. C. (1995). Periodic boundary conditions in ab initio calculations. *Physical Review B*, *51*(7), 4014.
